# Molecular profiling and computational network analysis of TAZ-mediated mammary tumorigenesis identifies actionable therapeutic targets

**DOI:** 10.18632/oncotarget.2570

**Published:** 2014-10-24

**Authors:** Costa Frangou, Ying-Wei Li, He Shen, Nuo Yang, Kayla E. Wilson, Maxime Blijlevens, Jin Guo, Norma J. Nowak, Jianmin Zhang

**Affiliations:** ^1^ Department of Cancer Genetics, Roswell Park Cancer Institute, Buffalo, NY, 14263, USA; ^2^ Department of Biochemistry, School of Medicine and Biomedical Sciences, University at Buffalo, The State University of New York, Buffalo, NY, 14260, USA

**Keywords:** breast cancer, TAZ, tumor-initiating cells, RNA sequencing, Dasatinib

## Abstract

Triple-negative breast cancer (TNBC) accounts for approximately 15–20% of all breast cancer (BC) cases and contributes disproportionately to BC mortality. TAZ, a key transducer of the Hippo pathway, has recently been demonstrated to confer breast cancer stem cell (CSC) traits. However, TAZ target genes and the underlying transcriptional regulatory pathways responsible for the CSC phenomenon remain unknown. Here, we demonstrate that the oncogenic activity of TAZ is essential for propagation of the malignant phenotype. We further show that constitutively active TAZ tumor-derived cells exhibit unique tumor-initiating properties, including increased self-renewal and metastatic seeding potential, acquired chemotherapy resistance and the ability to efficiently regenerate tumor formation *in vivo*. Combined digital RNA expression analysis and computational network approaches identify several signaling pathways that distinguish breast cancer tumor-initiating cells (T-ICs) from bulk tumor cells. We demonstrate the utility of this approach by repositioning the small molecule tyrosine kinase inhibitor, Dasatinib, which selectively targets T-ICs and inhibits TNBC growth *in vivo*.

## INTRODUCTION

The Hippo pathway is an evolutionary conserved regulator of tissue growth and cell fate during mammalian development and regeneration, while deregulation of the Hippo pathway caused by gene mutation or anomalous protein expression has been linked to human diseases including cancer [1, 2]. Currently, over 20 regulators have been identified that intersect with core Hippo pathway components and potentially contribute to carcinogenesis. For example, loss of Neurofibromin2 (NF2) tumor suppressor function has been associated with the development of colorectal, hepatocellular and thyroid carcinomas, as well as in melanoma [3]. Central to the Hippo pathway are two key downstream effector proteins, YAP (Yes-associated protein) and TAZ (transcriptional co-activator with PDZ-binding motif) that are tightly regulated by a number of upstream signaling molecules, such as Mst1/2, Lats1/2 and RASSF family proteins [4, 5]. Inactivation of YAP/TAZ by the Hippo pathway is mediated primarily *via* cytoplasmic sequestration from 14-3-3 binding and protein degradation, respectively [6–9]. In addition, Hippo pathway-independent restriction of YAP/TAZ mediated by angiomotin, scribble, PTPN14, α-catenin and other junction protein sequestration mechanisms has been reported [10–15].

TAZ does not contain an intrinsic DNA-binding domain, but instead actively recruited to target genes by interacting with multiple transcription factors and mediates diverse transcriptional programs in a context-dependent manner [16]. Interestingly, loss of the TAZ protein resulted in uncontrolled differentiation of human embryonic stem cells (hESCs) as well as loss of self-renewal of hESCs [17]. Furthermore, TAZ was recently shown to sustain self-renewal potential and tumor-initiation capacities of breast CSCs [11]; however, TAZ target genes and the underlying transcriptional regulatory pathways responsible for the CSC phenomenon remain poorly characterized. Nonetheless, therapeutic modulation of TAZ could improve current cancer treatment strategies.

Unfortunately, direct pharmacological inhibition of TAZ is challenging because it has no known catalytic activity [18, 19]. Accordingly, in the current study we employ a strategy that exploits the functional interconnectivity of intracellular signaling networks to unambiguously identify disease-specific ‘druggable’ targets, located downstream of TAZ. Moreover, we clearly demonstrate that expression of constitutively active TAZ experimentally confers BC T-IC properties and metastatic colonization capacity to non-transformed human basal-like mammary epithelial cells. Notably, we present a novel differential network-based framework to detect biologically meaningful cancer-related genes and subsequently prioritize/rank genes as potential drug targets. Finally, we demonstrate that Dasatinib, a Src family kinase and receptor tyrosine kinase (RTK) inhibitor, selectively targets TAZ-induced T-ICs and provides novel insight to how modulation of TAZ-driven pro-tumorigenic transcriptional programs could help guide future BC treatment strategies.

## RESULTS

### *In vivo*-derived TAZ-induced tumor initiating cells (T-ICs)

**Figure 1:**
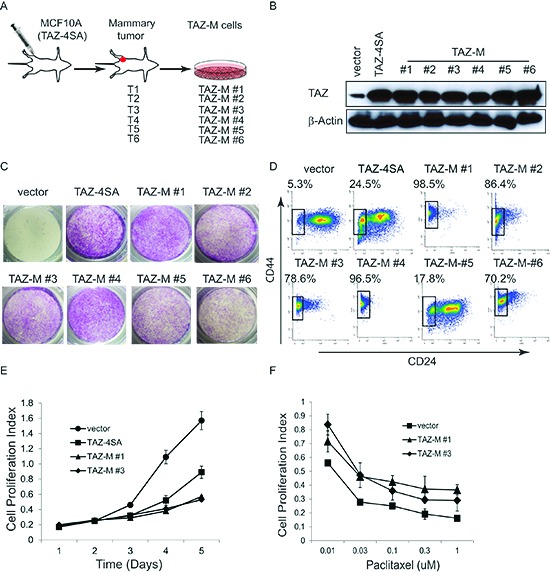
TAZ-induced mammary tumor derived cells exhibit a robust CSC potential **(A)** Schematic description of the establishment of TAZ-4SA-derived mammary tumors and explanted cell lines. **(B)** Immunoblot of TAZ in the tumor-derived cells as well as in the parental TAZ-4SA MCF10A cells. β-Actin was used as the loading control. **(C)** Cell migration is increased in the tumor-derived cells. **(D)** Flow cytometry of the CD44^high^/CD24^low^ population in the tumor-derived cells as well as in the vector control or TAZ-4SA transduced MCF10A cells. **(E)** Proliferation curve of vector or TAZ-4SA transduced MCF10A cells, as well as TAZ-M#1 and TAZ-M#3 cells grown in culture. Viable cells were counted by the MTT assay. Bars denote standard errors (n = 5). **(F)** Dose-response curve of vector-transduced MCF10A cells, TAZ-M#1 and TAZ-M#3 cells treated with paclitaxel. Bars denote standard errors (n = 5).

TAZ has been previously indicated to serve as a key breast CSC determinant [11]. Accordingly, to further characterize TAZ-induced CSC-like traits during mammary tumorigenesis, we orthotopically injected *TAZ-4SA* (constitutively activated form) transduced MCF10A cells into the mammary fat pad of SCID mice and generated primary tumors. We explanted the TAZ-induced mammary tumors and derived cell lines (herein denoted TAZ-M#1-6) that could be stably propagated in tissue culture (Fig. 1A). We confirmed ectopic TAZ expression in all tumor-derived cells as well as in the parental cell line by immunoblot (Fig. 1B). We previously showed that overexpression of constitutively active TAZ promoted cell migration [20]. To independently test whether tumor-derived cell lines maintained such capacity, we performed transwell cell migration assays and found that they all migrated aggressively (Fig. 1C).

A subpopulation CD44^high^/CD24^low^ of breast cancer cells has been reported to have stem/progenitor cell properties [21]. To estimate the proportion of CD44^high^/CD24^low^ cells, we characterized tumor-derived cell lines by flow cytometry for surface expression of CD44 and CD24, respectively. As shown in Figure 1D, this population was indeed enriched in the majority of tumor-derived cell lines tested but phenotypically reverted to the CD44^high^/CD24^high^ population after extended culture (Supplemental Fig. 1A). Interestingly, the CD44^high^/CD24^low^ sub population displayed a lower *in vitro* proliferation rate compared to parental cells and resistance to chemotherapy (Fig. 1E, F). However, they exhibited selective sensitivity to Salinomycin [22] and Bortezomib [23] (Supplemental Fig. 1B, C), consistent with the recently reported proteasome addiction of basal-like TNBC cells [23].

**Figure 2:**
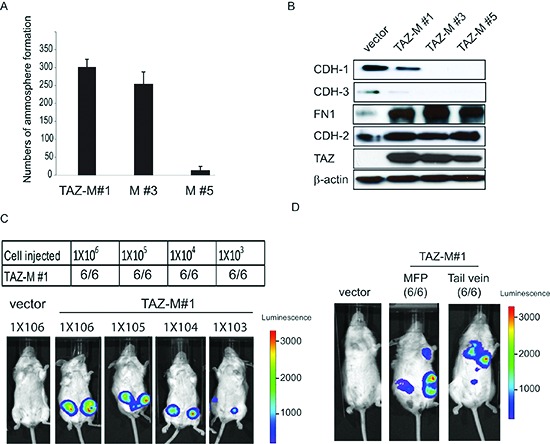
Tumor-derived cells acquired efficient tumor initiation and metastatic capacity **(A)** Quantification of mammosphere formation in TAZ-M#1, TAZ-M#3 and TAZ-M#5 cells. Bars denote standard errors (n = 5). **(B)** TAZ-M#1, TAZ-M#3 and TAZ-M#5 cells undergo EMT. Immunoblot shows decreased E-cadherin and P-cadherin (epithelial markers) and concomitantly increased N-cadherin and fibronectin (mesenchymal markers). β-Actin was used as the loading control. **(C)** Tumor-seeding capacity of TAZ-M#1 cells when injected into the mammary fat pad of NOD/SCID mice. **(D)** Metastatic tumor-seeding capacity of TAZ-M#1 cells when injected into the mammary fat pad and tail vein of the NSG mice.

Next, to determine whether the tumor-derived cells contained self-renewal capacity, we used an *in vitro* mammosphere formation assay and found that majority of the cell lines generated mammospheres of increased size and number (Fig. 2A). The acquisition of CSC traits has been previously associated with the epithelial to mesenchymal transition (EMT) [24]. Therefore, we further analyzed the alterations of EMT markers in the tumor derived cells. The alterations of EMT-associated markers, such as: loss of epithelial genes CDH1, CDH3 and gain of mesenchymal genes CDH2 and FN1, as well as the adoption of a migratory mesenchymal phenotype were maintained in all the tumor-derived cell lines (Fig. 1C and 2B). Interestingly, the TAZ-M#5 cell line underwent EMT but lost the stem-like features (Fig. 1C&D, 2 A&B) and tumor initiation potential (data not shown).

To explore whether tumor-derived CD44^high^/CD24^low^ cells acquired tumor-initiating capacity and to accurately enumerate their frequency, we used *in vivo* clonal tumor initiating assays to functionally identify CSCs rather than on the basis of immunophenotype or *in vitro* mammosphere assay. Consequently, we estimated a > 1000-fold increase in the frequency of self-renewing T-ICs in the TAZ-M#1 cell line relative to the parental TAZ-4SA-expressing MCF10A cell line (Fig. 2C and data not shown). The likelihood of nodal metastases is increased in BC patients whose tumors have breast CSCs [21]. Furthermore, emerging evidence indicates that breast CSCs and EMT co-operate to generate circulating tumor cells (CTCs) that are highly competent for metastasis [25]. To further explore this possibility and specifically determine whether the tumor-derived cells promote metastasis, both mammary fat pad and tail-vein injections were performed in NSG mice. In contrast to control mice, both metastasis to the lungs and spontaneous metastasis were observed in NSG mice injected with tumor-derived TAZ-induced cells (TAZ-M#1), strongly suggesting that TAZ increased metastatic potential and tumor-seeding ability (Fig. 2D).

### Network-based comparison of TAZ-mediated gene expression patterns

**Figure 3:**
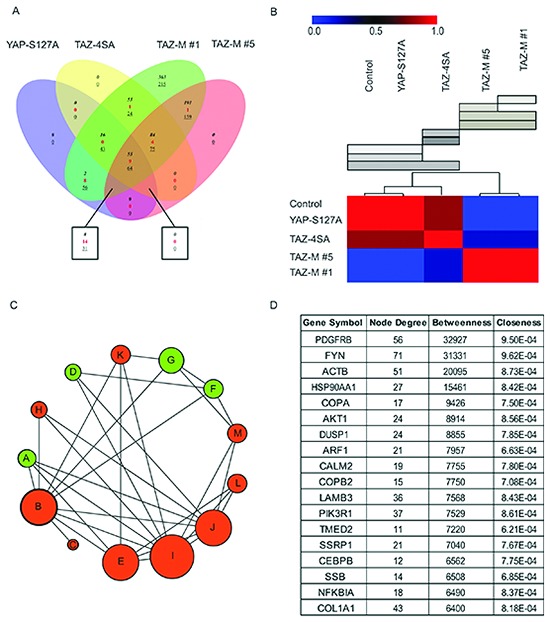
Identification of common and distinct TAZ target gene signatures **(A)** Venn diagram describing the number of genes that changed in each condition and their relation to each other. To indicate the polarity of numerical regulation of each factor within each set or intersection, we have employed a three-way key system. The numbers of factors unique to a set, with a positive regulation polarity, are identified with an italic numeral. The numbers of factors unique to a set, with a negative regulation polarity, are identified with an underlined numeral. The numbers of factors common between multiple sets are indicated with a red colored numeral. **(B)** TAZ-M#1 and TAZ-M#5 tumors clustered using NMF expression signatures from RNA-Seq data sets and the consensus clustering matrix for the clusters are shown. Based on the visual inspection of a hierarchical clustering of the consensus matrix defining the average connectivity over 1000 clustering runs with different initial conditions, the case of K = 5 was used to arrange samples in, giving rise to 5 clusters highlighted by red and blue, respectively. **(C)** Graphical depiction of enriched pathway modules obtained by edge-betweenness network clustering results for the altered genes from the TAZ-M#1 and TAZ-M#5 functional interaction (FI) networks. Each node is a pathway and nodes are linked by directed edges representing parent-to-child relationships. Nodes are colored by red (common networks) or green (TAZ-M#1 specific); the size of each node is proportional to the number of proteins annotated with that term. **(D)** Table summarizing the degree and centrality measures for the top 18 TAZ-M#1 bottlenecks proteins that are defined as central nodes. The bottlenecks control the flow of biological information within the TAZ-M#1 network, and their disruption can break the entire network into small components. From 18 major bottlenecks observed within the network, 5 bottlenecks represent new protein targets that are suitable for the development of TNBC.

To examine the TAZ-mediated pro-tumorigenic transcriptional program, we performed RNA-seq differential gene and transcript analysis for YAP or TAZ transduced MCF10A cells, as well as a representative subset of tumor-derived cell lines (TAZ-M#1 and TAZ-M#5). Comparison of the expression profiles identified 1478 genes that displayed a significant difference between samples (Fig. 3A and Supplemental Table 1). As shown in Figure 3B, five data subtypes were identified by consensus NMF clustering using an abundance matrix containing differentially changed genes (> 2.0 fold change, with p < 0.05 in all expression profiles). Using DAVID (database for annotation, visualization and discovery), we assessed whether any GO biological or molecular processes were statistically over- or under-represented. Over-represented pathways (FDR < 0.001) included EMT related pathways, along with integrin signaling and focal adhesion, chromatin remodeling, PIK3 signaling, growth hormone and nuclear receptor co-activators, WNT/cadherin signaling and ATM/Rb related pathways (Supplemental Table 2–4). In addition, Gene Set Enrichment Analysis (GSEA) of differentially regulated transcripts in the TAZ-M#1 gene cluster/signature revealed an overrepresentation of genes associated with several published invasive, metastatic BC patient and breast CSC transcriptome studies (Supplemental Table 5).

To expand on these analyses, we constructed a functional interaction (FI) network and used network community analysis to identify sub-networks that contain modules involved in annotated biological processes and canonical pathways [26]. Specifically, two interaction networks were independently generated from the TAZ-M#1 and TAZ-M#5 tumor data sets. Network subtraction was subsequently used to obtain a differential-network model and to identify both overlapping and unique clusters (Fig. 3C and Supplemental Table 6). Network comparison reflected differential functional interactions unique to TAZ-M#1, a breast tumor that had acquired stem-like traits. PDGFR-β, WNT and NF-kappa B signaling pathways were highly deregulated in TAZ-M#1 (FDR < 0.0001), and may represent key mediators and/or regulators of biological properties associated with CSCs [27].

Next, to reduce the list of candidate genes to those that are most likely associated with signaling pathways involved in self-renewal and activated in CSCs, as opposed to general cell proliferation and pro-survival mechanisms, we identified all network bottleneck proteins. These proteins are known to control the flow of biological information within a network and maintain network integrity. Briefly, we define network bottlenecks as nodes (proteins) with the highest betweenness centrality scores and estimated by calculating the number of shortest paths that cross a given node. In addition, highly connected nodes (hubs) are also known to play an integral role in the propagation of signals across a network and were also calculated (Fig. 3D & Supplemental Methods). As summarized in Fig. 3D, top-ranked bottleneck genes are highly deregulated in BC patients and several genes, such as PDGFR-β, FYN and NF-kappaB components.

### Dasatinib targets the TAZ-induced T-ICs and TNBC Cells

**Figure 4:**
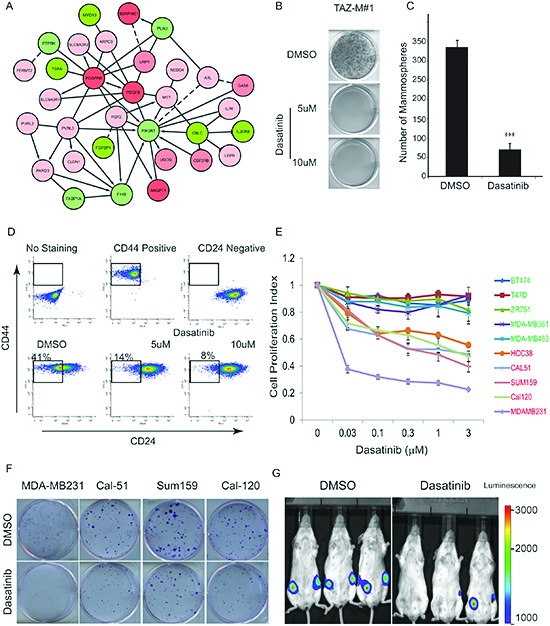
Identification of Dasatinib as a potent inhibitor for TAZ-induced stem-like properties and TNBC tumorigenicity **(A)** PDGFR-β-centered subcluster identified by the edge-betweenness algorithm. Node colors represent relative expression levels on a gradient scale ranging from green (low) to red (high). The magnitude of the gene change is proportional to the darkness of the color. Solid line: functional interactions (FIs) extracted from pathways; dashed line: based on naïve-bayes classifier (NBC); arrow: FIs involved in activation, expression regulation or catalysis; ‘T’ bar: FIs involved in inhibition. **(B)** Colony formation of TAZ-M#1 cells in soft agar is inhibited by Dasatinib. (n = 6) **(C)** Quantification of mammosphere formation induced by TAZ-M#1 cells treated in the DMSO vehicle or Dasatinib (1μM) for 6 days. Bars denote standard errors (n = 6) (***p < 0.0001). **(D)** FACS analysis of the CD44^high^/CD24^low^ population of TAZ-M#1 cells treated with either vehicle or Dasatinib at indicated doses for 24 hours and followed by recovery for 72 hours. **(E)** Dose-response curve of luminal (in green) and TNBC (in red) cell lines treated with Dasatinib for 72 hr. **(F)** Colony formation assays of cell lines treated with Dasatinib (300 nM) for 24 hr and cultured for 10 days in drug-free medium. **(G)** Tumor formation of MDA-MB-231 cells is inhibited by Dasatinib treatment. Cells were treated with DMSO or Dasatinib (300 nM) for 24 hr prior to mammary fat pad injection into the SCID mice.

Data presented thus far identify TAZ as a potential new drug target to treat aggressive types of BC. Unfortunately, no pharmacological agents directly targeting active TAZ have been reported. Therefore, we rationalized that modulating ‘druggable’ targets downstream of TAZ, rather than the oncogene itself, could be therapeutically exploited. Therefore, we used a subtractive integrated network approach and generated a TAZ-specific bipartite graph in which every link connected all FDA-approved drugs to a protein, if the protein is a known target of the drug (i.e. drug-target network, DTN) (Supplemental Table 7). As summarized in Figure 4A, one FI sub-cluster of particular interest was highly enriched with the PDGFR-β signaling pathway components. Furthermore, PDGFR-β, FYN and Src genes included in this module were independently identified as key bottleneck network genes (Fig. 3D).

Dasatinib is a potent, multi-target kinase inhibitor predicted from our DTN analyses (Supplemental Table 7). It was initially developed as an inhibitor of the Src family kinases (SFKs, such as Fyn, Yes, Src and Lyk) but is now known to also inhibit BCR-ABL, EphA2, platelet derived growth factor receptor (PDGFR) and c-KIT [28]. Accordingly, we rationalized that Dasatinib would represent an apposite and novel multi-target repositioning agent to pharmacologically modulate TAZ-mediated pro-tumorigenic transcriptional programs. To our great interest, we found that Dasatinib inhibited the anchorage-independent growth of TAZ-M#1 cells in soft-agar assay and reduced self-renewal as measured by mammosphere formation (Fig. 4B, C). Remarkably, FACS analysis of Dasatinib-treated TAZ-M#1 cells had an almost completely depleted CD44^high^/CD24^low^ subpopulation after drug treatment for 24 hours and recovery of 72 hours; and only the CD44^high^/CD24^high^ BC subpopulation remained viable (Fig. 4D). Collectively, these results indicate that Dasatinib selectively killed the TAZ driven BC T-ICs.

TNBC frequently has enriched breast CSCs that are thought to drive tumorigenesis and contribute to maligancy, therapeutic resistance and clinical relapse [29]. To further explore whether Dasatinib specifically targets TNBC cells, we used a panel of human TNBC and luminal cell lines to further assess differential sensitivity and response to Dasatinib. First, we examined the half-maximal inhibitory concentration (IC50) values for Dasatinib and found that TNBC cell lines were more sensitive to Dasatinib than luminal cell lines (Fig. 4E). In addition, we confirmed the effect of Dasatinib on TNBC cell line proliferation *in vitro* by colony formation assay (Fig. 4F). Finally, to evaluate the effect of Dasatinib on mammary tumor-forming potential *in vivo*, we injected either vehicle control or 24-hour low-dose (300 nM) Dasatinib treated MDA-MB-231 cells into the mammary fat pad of SCID mice and clearly demonstrated that Dasatinib decreased tumor formation capacity (Fig. 4G).

## DISCUSSION

The CSC hypothesis is based on the observation that many cancers are driven by a subpopulation of tumor-initiating cells (popularly known as CSCs) and promote tumor growth. In addition, CSCs are resistant to chemotherapy and radiation treatments, which potentially explain the limitations of these agents in curing human malignancies [30–32]. Despite considerable research efforts in recent years, it remains unclear what fraction of cancers follow the CSC model as well as the clinical behaviors explained by the model. Nonetheless, delineation of essential pathways that can distinguish T-ICs from their normal counterparts could provide novel opportunities for therapeutic intervention.

Multi-omics approaches allow the characterization of biological systems in various disease states to be measured from an increasing number of biochemical and molecular perspectives. Unfortunately, conventional functional enrichment analyses are limited in their efficacy because they do not incorporate known interdependencies among genes within a pathway. Furthermore, they treat all gene alterations as equal, which is not expected to be valid for many biological systems. Conversely, network-based pathway analysis identifies markers not as individual entities but as sub-networks and the resulting sub-networks provides an effective way to visualize genes and their interactions/relationships [33]. Here, employing digital RNA sequencing and computational approaches, we present a differential network-based framework to detect biologically meaningful cancer-related genes.

Notably, we identify several developmental pathways currently under investigation as potential therapeutic targets in CSCs, such as the Hedgehog, WNT, NOTCH and Nodal/Activin pathways [34]. However, at present, it is unclear of which these pathways are direct or indirect targets of a TAZ-mediated pro-tumorigenic transcriptional program. Nevertheless, our network analyses identified a novel PDGFR-β centered FI sub-cluster. The role of the PDGFR-β in different tumor cell types and in metastasis remains poorly characterized. For example, PDGFR-β signaling contributes to diverse tumor-associated processes, such as autocrine growth simulation of malignant cells, paracrine-stimulation of blood vessel formation and stimulation of fibroblasts and pericytes in the tumor stroma [35]. It was recently reported that mutant p53-induced up-regulation of PDGFRβ drives pancreatic cancer invasion and metastasis [36]. Of the intra-cellular pathways that operate downstream of PDGFR-β, the activation of the Ras and ERK MAP kinase pathway is important for cell proliferation. Actin reorganization and cell migration is dependent on PI3-kinase mediated activation of Rac, whereas PI3-kinase activation of AKT is important for cell survival. Several of these pathways are up- regulated in TAZ-expressing tumors as determined by high-density antibody microarrays (data not shown). However, direct inhibition of PDGFR-β alone through either RNAi or PDGFR-specific inhibitors, had no effect on BC cell viability *in vitro* or TAZ-mediated tumorigenicity *in vivo* (data not shown).

Recent advances in computational biology have identified a network structure and phenotypic robustness that strongly suggests the higher clinical efficacy of multi target drugs as compared with individual drug targets. Adopting this basic tenet, we rationalized that combinatorial gene inhibition by the tyrosine kinase inhibitor such as Dasatinib could modulate TAZ-mediated tumorigenesis. Correspondingly, we clearly show that Dasatinib selectively targets the CD44^high^/CD24^low^ subpopulation *in vitro* and inhibits TNBC tumor growth *in vivo.* In a recent study, PDGFR-β was shown to lie downstream of FOXC2 in BC cells induced to undergo EMT. Furthermore, the PDGFR-β inhibitor sunitinib reduced tumor growth and metastasis of FOXC2-expressing tumor cells [37], but given our findings it is most likely the effects mediated by sunitinib in these studies are a consequence of complex RTK inhibition and not direct PDGFR-β inhibition.

Current failure in TNBC treatment usually is not due to a lack of primary response or initial induction of remission but instead relapse after chemotherapy, in which CSCs are believed to play an essential role. If the CSC hypothesis is correct, then the most appropriate cells for investigation of genes involved in TNBC may be CSCs. Here, we demonstrate how the multi-target inhibitor, Dasatinib, selectively targets CSCs and inhibits basal-like and TNBC tumor growth. Similar to other targeted cancer therapies it will be important to determine which BC patients are most likely to benefit from this treatment. Furthermore, future studies are required in defining optimal treatment strategies for aggressive TNBC, both in the advanced as well as adjuvant setting.

## MATERIALS AND METHODS

### Cell culture, transfection and transduction

MCF10A cells were cultured as described [38]. MDA-MB-231, HCC38, T47D and ZR751 cells were cultivated in RPMI-1640 medium with 10% fetal calf serum (FBS); CAL120, MDA-MB 361, MDA-MB 453 and BT474 cells were cultivated in DMEM medium with 10% FBS; CAL 51 was cultivated in DMEM medium with 20% FBS; SUM159 was cultivated in F12K medium with 5% FBS, 5 μg/ml insulin and 1 μg/ml hydrocortisone. All media were supplemented with 100 units/ml penicillin, 100 μg/ml streptomycin and 2 mM glutamine. All cells were cultured in a humidified atmosphere of 95% air and 5% CO2 at 37ºC. MDA-MB-231, T47D, MDA-MB453, BT-474 and HCC38cells were purchased from American Type Culture Collection (ATCC, VA); Cal51, Cal120 and SUM159 were obtained from Dr. Toru Ouchi, PRCI; ZR751 and MDA-MB-361 were obtained from Dr. Andrei V. Bakin, RPCI. Transfection was performed using X-tremeGENE 9 DNA Transfection Reagent following the manufacturer's protocol (Roche). Packaging of retrovirus and lentivirus, cell transduction and drug selection were performed following standard protocols.

### Plasmid construction

The human *TAZ-4SA* expression constructs were described previously [20].

### Antibodies and immunoblot analysis

TAZ antibody was purchased from Cell Signaling Technology; YAP antibody from Santa Cruz; β-actin antibody from Upstate; fibronectin and Flag (M2) antibodies from Sigma-Aldrich; E-cadherin (CDH1), P-cadherin (CDH3), N-cadherin (CDH2), FN1 antibodies from BD Biosciences; CD24-PE and CD44-APC antibodies from Invitrogen. For protein extraction, cells were washed with phosphate-buffered saline (PBS) and collected with IP buffer: 20 mM Tris-HCl (pH 8.0), 150 mM NaCl, 20% glycerol, 0.5% NP-40, plus 1 ´ Complete^TM^ EDTA-free Protease Inhibitor Cocktail (Roche) or 1 ´ Halt^TM^ EDTA-free Protease and Phosphotase Inhibitor (Thermo Scientific). Cell lysate was cleared by centrifugation at 14,000 rpm for 20 min at 4°C. Lysate was loaded onto 4–15% MINI-PROTEAN TGX gel (Bio-Rad) with 4X SDS sample buffer. For immunoblot, proteins were transferred onto Immobilon-P membrane (Millipore), detected by various antibodies and visualized with ECL Plus Western Blotting Detection Reagents (GE Healthcare).

### Flow cytometry analysis

Cells were passed through a 35-μm filter, pelleted, washed in 1X phosphate buffered saline (PBS) + 0.5% fetal calf serum (FBS) and counted. One million cells were suspended in 1X PBS + 0.5% FBS and stained with anti-CD44-APC conjugate and anti-CD24-PE conjugate (BD Biosciences) for 30 min at 4°C. Cells were washed 3 times and then analyzed by flow cytometry.

### Mammosphere formation assay

Mammosphere formation assay was performed by plating 5×10^4^ cells in serum-free DMEM/F12 1:1 media (Gibco) supplemented with EGF (20 ng/mL) and B27 (2%) into ultra-low attachment 6-well plates (Corning). Mammospheres were allowed to grow or in presence of drugs for 7 days. Total mammospheres greater than 100 μm in diameter were counted. Each experimental group was done in triplicate and the same experiments were repeated at least three times.

### Cell proliferation (MTT) assay

Two thousand to four thousand cells were plated into 96 wells plate. Plates were harvested daily. Twenty microliters of 5mg/ml MTT (3-(4, 5-Dimethylthiazol-2-yl)- 2, 5-diphenyltetrazolium bromide, a tetrazole) was added to each well, incubated for 3.5 hours in 37°C, and carefully removed without disturbing the cells. One hundred and fifty microliters of MTT solvent (4 mM HCl and 0.1% Nondet P-40 (NP40) in isopropanol) was added into each well and plate shaken at room temperature for 15 min. Absorbance was read at 590 nm with a reference filter of 620nm.

### Anchorage-independent growth

Colony formation in soft agar assay was performed as described previously [10]. Briefly, cells were suspended in a growth medium mixed with 0.4% agar and seeded into 6-well plate containing a base of 0.5% agar, at a concentration of 5×10^4^ cells/well. Cells were incubated and colonies were read after approximately 2 weeks.

### *In vivo* tumor growth and metastasis assays

For TAZ tumor formation, 1 ´ 10^6^ TAZ-4SA transduced MCF10A were injected into the mammary fat pad of female NOD/SCID mice of 6–8 weeks old. For serial dilution experiments, vector control or TAZ-4SA transduced MCF10A cells, TAZ-M#1 or TAZ-M#5 cells in exponential growth phase were harvested and suspended in PBS (50% matrigel), and 1 × 10^6^ (in 0.1 mL) vector or 1 × 10^6^, 1 × 10^5^, 1 × 10^4^, 1 × 10^3^ TAZ-4SA transduced MCF10A, TAZ-M#1 and TAZ-M#5 were injected into the mammary fat pad of female NOD/SCID mice of 6–8 weeks old. The SCID mice were generated at the Roswell Park Cancer Institute. Tumor sizes were measured twice a week using calipers. Mammary tumor formations were also detected by the *In Vivo* Luminescence Imaging System. For tail vein injection, 1 × 10^6^ (in 0.1 mL) TAZ-M#1 cells were injected into the tail vein of female NSG mice of 6–8 weeks old. Lung tumor formations were detected by the *In Vivo* Luminescence Imaging System. The care and use of animals was approved by the Institutional Animal Care and Use Committee of the Roswell Park Cancer Institute (Buffalo, NY).

### Cultivation of explanted tumor cells

An explant method was used for culturing mouse tumor cells adopted from previous description [39]. Briefly, the primary mouse mammary tumors were excised, washed with growth medium (MCF10A growth medium) and then minced with scalpels to obtain pieces approximately 2 mm in diameter. Twelve to fifteen of these pieces were then evenly dispersed over the bottom of each 6-cm dish (Falcon Plastics) and placed at room temperature for approximately 15 minutes to allow attachment. The tissue was then covered with 5 ml MCF10A growth medium. Cultures were incubated at 37ºC and observed daily for evidence of growth. During the first week, the culture medium was changed only if a large amount of cellular debris was in the flasks or if the pH became noticeably alkaline or acidic. Care was taken to avoid dislodging the tissue explants. After the first week or after significant growth, the medium was changed at least twice a week. When cultures were confluent, cells were conservatively sub-cultured.

### Integrative informatics and pathway analysis

For network and pathway enrichment analysis, we used the Reactome FI and Cytoscape plugin. Significantly enriched pathway gene sets were exported and analyzed using Enrichment Map to determine relationships between pathways [40]. To investigate functional network and gene ontology relationships, candidate genes were first categorized using the GO analysis and were performed with the DAVID software [41]. The reference population was defined by our gene study set (see above) and an adjusted P value of 0.05 (Benjamini and Hochberg correction was used as a threshold for the identification of significant GO terms.

## SUPPLEMENTARY METHODS, FIGURE AND tables


