# Supplementary material for: Molecular profiling and computational network analysis of TAZ-mediated mammary tumorigenesis identifies actionable therapeutic targets
Source: Oncotarget. 2014 Oct 24;5(23):12166–76. doi: 10.18632/oncotarget.2570 (PMC4323005; doi:10.18632/oncotarget.2570)
Supplement: Supplementary file 1 [file oncotarget-05-12166-s001.pdf]

SUPPLEMENTARY METHODS, FIGURE AND TABLES

Transcriptome sequencing

cDNA synthesis, library preparation of six indexed samples and RNA-Seq analysis was performed at the AGRF on an Illumina HiSeq2000 to a minimum depth of >25 million single reads of 50bp. Raw reads were assessed for good quality using the FASTQC software. Alignment of transcript sequences to the human reference genome (build hg19) was performed using the TopHat software with default parameters [1]. Gene regions were identified based on alignments of the RefSeq human database by the UCSC genome browser (hg19).

Gene and transcript annotations used in the analyses correspond to those in GENCODE v11. We focused on protein coding genes and filtered out those for which at least one of the annotated transcripts was shorter than 300 bp, given that those transcripts would be lost during the size selection step in the RNA-seq experiment. In total, our study set comprises 18,450 protein coding genes, of which 14,902 have more than one transcript annotated.

Exonic coordinates were retrieved from the annotation and used to define intronic regions. Formally, our definition of intron encompasses those regions that are located inside genic boundaries and are not overlapped by any exon in any annotated transcript. We then computed the number of reads overlapping known exons and introns using dexseq-count (DEXSeq v1.5.5) [2] and were calculated by counting aligning reads per kilobase per million reads mapped (RPKM). Absence of gene expression was defined as an RPKM value of <1. RNA-SEQ. For each gene, expression levels were calculated as the average RPKMs of all expressed exons. Independently, transcript abundances were obtained using three different tools: MISO [3], Cufflinks v1.3.0 [4] and MMSEQ [5]. MISO and Cufflinks take as input alignments to the genome, while MMSEQ requires mapping to the transcriptome, thus the need to use two different mapping strategies. In all three cases we based the estimates on the existing transcript annotation, cancelling any option for de novo inference, and converted those to transcript relative abundances when necessary. In this manuscript we are referring to the results obtained by MISO and we use a default FPKM threshold of 1 to consider a gene/transcript as expressed.

De novo transcript discovery using cufflinks

We focused on those genes for which all of the annotated transcripts can be uniquely identified by at least one splice junction. We then proceeded to identify major transcripts based on coverage evidence (that is, quantifying the number of reads supporting each junction and taking the average in case of several splice junctions). For each sample we calculated the overlap with MISO.

We used Cufflinks to discover novel transcripts in each sample data set and merged all the obtained annotations using cuffmerge. We then focused on the subset of transcripts that overlap with known protein coding genes and filtered out those genes with transcripts shorter than 300bp.

Functional interaction network construction

The functional interaction network used in this study was described in Wu *et al. [6]*. Briefly, we compiled protein pairwise relationships extracted from protein-protein interactions from human, yeast, worm and fly, gene co-expression data sets, Gene Ontology annotations, domain-domain interactions, and text-mined protein interactions. A Bayes classifier was used to predict functional interactions for protein pairs, and the predicted FIs merged with FIs extracted from curated pathways in Reactome [7] , KEGG [8], NCI-PID [9], Panther [10] and CellMap (http://cancer.cellmap.org/cellmap).

We obtained the protein-protein interaction network from the Human Protein Reference Database. This network contained 9,667 proteins and 76,132 binary edges. We obtained KEGG, Biocarta, and Reactome gene sets from MsigDB [11] and all conserved subnetworks in the human protein-protein interaction network from [12]. To reduce bias to disease proteins in the protein-protein interaction network, we use the extended protein-protein interaction network suggested by [13]. The extended protein-protein interaction network is generated by combining the HPRD, OPHID, BIND and MINT database, and has a similar degree of interactions for both disease and non-disease proteins.

MCL network clustering

We chose MCL (http://www.library.uu.nl/digiarchief/dip/diss/1895620/inhoud.htm) as the network-clustering algorithm in order to take advantage of edge weights. We weighted each interaction edge according to the absolute value of the PCC of the expression levels of the two genes connected by the edge. To control the size of network modules generated from the MCL clustering, we used 6.0 as the inflation coefficient and average PCC >0.27. For permutation testing, we randomly swapped expression values for all genes, or randomly selected genes from the FI network.

Cancer gene enrichment analysis

The cancer gene enrichment analysis examines over-representation of known cancer genes in a gene signature. Assuming the total number of genes ***N***, cancer genes ***M***, and signature genes ***J***, the probability of having more than ***K*** cancer genes in a signature follows a hypergeometric distribution:

$$P\ (\#\text{ of cancer genes} > K) = 1 - \sum Ki = 0\ (Ji)\ (N-JM-i)(NM).$$

Significance of pathway scores

To assess significance of the pathway score, we performed the analysis on random datasets. To construct these control datasets, we randomly shuffled initial gene activity scores and pathway member assignments 10,000 times to generate a background distribution of pathway scores. From this control dataset we were able to derive the empirical p-value of the actual scores.

Pathway gene ranking

In this study, we utilize "gene ranking" or the relative ordering of the genes based on their expression levels within each profile [14, 15]. Consider a pathway that contains $\boldsymbol{n}$ member genes $\boldsymbol{G} = \{\boldsymbol{g_1}, \boldsymbol{g_2}, \ldots, \boldsymbol{g_n}\}$ after removing the genes that are not included in all datasets. Given a sample $x_k = \{\mathbf{x_k}^1, \mathbf{x_k}^2, \ldots, \mathbf{x_k}^n\}$ that contains the expression level of the member genes, the gene ranking $\mathbf{r_k}$ is defined as follows:

$$\mathrm{r}k = \{ri, jk \mid 1 \le i < j \le n\}, \qquad \text{[i]}$$

where

$$ri, jk = \{1, 0, \text{ if } xik < xjk, \text{ otherwise}, \qquad \text{[ii]}$$

The resulting gene ranking r_k is a binary vector representing the ordering of the member genes based on their expression values in the kth sample x_k. To preserve the gene ranking in each sample, we do not employ any between-sample normalization.

SUPPLEMENTARY REFERENCES

1. Trapnell C, Pachter L, Salzberg SL. TopHat: discovering splice junctions with RNA-Seq. Bioinformatics. 2009; 25:1105–11
2. UniProt C. Reorganizing the protein space at the Universal Protein Resource (UniProt). Nucleic acids research. 2012; 40:D71–5
3. Katz Y, Wang ET, Airoldi EM, Burge CB. Analysis and design of RNA sequencing experiments for identifying isoform regulation. Nature methods. 2010; 7:1009–15
4. Trapnell C, Williams BA, Pertea G, Mortazavi A, Kwan G, van Baren MJ, et al. Transcript assembly and quantification by RNA-Seq reveals unannotated transcripts and isoform switching during cell differentiation. Nature biotechnology. 2010; 28:511–5
5. Turro E, Su SY, Goncalves A, Coin LJ, Richardson S, Lewin A. Haplotype and isoform specific expression estimation using multi-mapping RNA-seq reads. Genome biology. 2011; 12:R13
6. Wu G, Feng X, Stein L. A human functional protein interaction network and its application to cancer data analysis. Genome biology. 2010; 11:R53
7. Croft D, O'Kelly G, Wu G, Haw R, Gillespie M, Matthews L, et al. Reactome: a database of reactions, pathways and biological processes. Nucleic acids research. 2011; 39:D691–7
8. Kanehisa M, Goto S, Kawashima S, Okuno Y, Hattori M. The KEGG resource for deciphering the genome. Nucleic acids research. 2004; 32:D277–80
9. Schaefer CF, Anthony K, Krupa S, Buchoff J, Day M, Hannay T. PID: the Pathway Interaction Database. Nucleic acids research. 2009; 37:D674–9
10. Mi H, Dong Q, Muruganujan A, Gaudet P, Lewis S, Thomas PD. PANTHER version 7: improved phylogenetic trees, orthologs and collaboration with the Gene Ontology Consortium. Nucleic acids research. 2010; 38:D204–10
11. Subramanian A, Tamayo P, Mootha VK, Mukherjee S, Ebert BL, Gillette MA, et al. Gene set enrichment analysis: a knowledge-based approach for interpreting genome-wide expression profiles. Proceedings of the National Academy of Sciences of the United States of America. 2005; 102:15545–50
12. Suthram S, Dudley JT, Chiang AP, Chen R, Hastie TJ, Butte AJ. Network-based elucidation of human disease similarities reveals common functional modules enriched for pluripotent drug targets. PLoS computational biology. 2010; 6:e1000662
13. Wu X, Jiang R, Zhang MQ, Li S. Network-based global inference of human disease genes. Molecular systems biology. 2008; 4:189
14. Geman D, d'Avignon C, Naiman DQ, Winslow RL. Classifying gene expression profiles from pairwise mRNA comparisons. Statistical applications in genetics and molecular biology. 2004; 3:Article19
15. Su J, Yoon BJ, Dougherty ER. Accurate and reliable cancer classification based on probabilistic inference of pathway activity. PloS one. 2009; 4:e8161

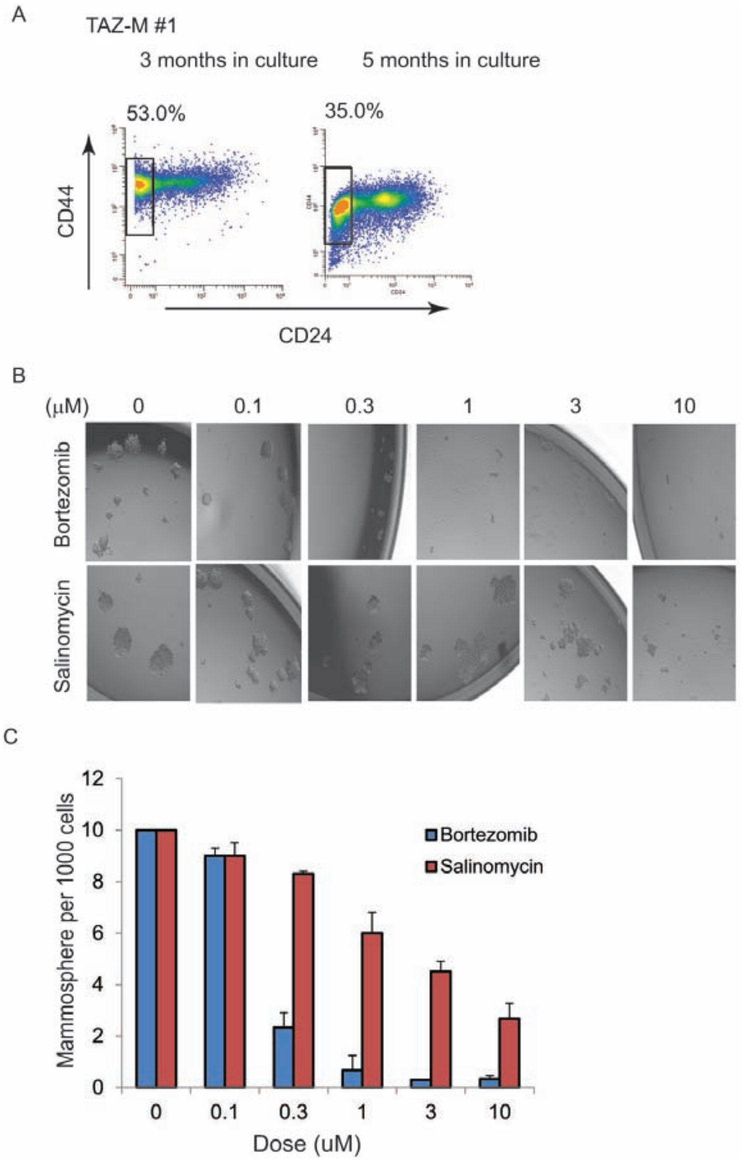

Supplementary Figure S1: Explanted TAZ-4SA-induced tumor cells display different sensitivity to Bortezomib and Salinomycin. **(A)** Flow cytometry analysis of the CD44^{high}/CD24^{low} population of the TAZ-M#1 cells at 3- and 5-month culture. **(B)** Representative images of mammophere formation of TAZ-M#1 in response to Bortezomib and Salinomycin. **(C)** Quantification of mammosphere formation of TAZ-M#1 in response to Bortezomib and Salinomycin. Bars denote standard errors (n = 3).

Supplementary Table S1: Data of RNAseq and Venn Diagram.

Supplementary Table S2: TAZ-M#1 and TAZ-M#5 overlapping pathways.

Supplementary Table S3: TAZ-M#1 specific pathways.

Supplementary Table S4: TAZ-M #5 specific pathways.

Supplementary Table S5: GSEA Modules.

Supplementary Table S6: TAZ-M #1, 5 Network model.

Supplementary Table S7: TAZ-M#1 drug-target network (DTN).
